# Association between sleep duration and albumin in US adults: a cross-sectional study of NHANES 2015–2018

**DOI:** 10.1186/s12889-022-13524-y

**Published:** 2022-06-02

**Authors:** Jingxian Li, Lizhong Guo

**Affiliations:** 1 Nanjing University of Chinese Medicine, Nanjing, 210029 China; 2 Jining NO.1 People’s Hospital, Jining, 272000 China

**Keywords:** Albumin, Sleep duration, Sleep timing, NHANES

## Abstract

**Background:**

Albumin has multiple functions and is used in the clinical assessment of liver function, kidney function and nutritional status. However, few epidemiological studies have evaluated the association between sleep duration and albumin. Therefore, we carried out a cross-sectional study to address this issue. The aim of the study was to investigate the association between sleep duration and albumin in American adults based on the NHANES (National Health and Nutrition Examination Survey)**.**

**Methods:**

A total of 9,973 participants aged $\ge$ 20 years were included in this study from NHANES 2015–2018. Weighted data were calculated according to analytical guidelines. Linear regression models and smooth curve fitting were used to assess and describe the relationship between sleep duration and albumin. The inflection point was determined by a two-step recursive method. Moreover, univariate and stratified analyses were performed.

**Results:**

There was an inverted U-shaped association between sleep duration and albumin levels. Albumin levels were highest when the sleep duration was 7.5 h. Compared to 7–8 h of sleep, short sleep duration was linked to lower albumin levels [sleep duration $\le$ 5 h: β $=$-1.00, 95% CI (-1.26, -0.74), *P* < 0.0001]. Compared to 7–8 h of sleep, long sleep duration was related to lower albumin levels [sleep duration $>$ 9 h: β $=$ -0.48, 95% CI (-0.68, -0.27), *P* < 0.0001].

**Conclusions:**

Sleep duration had an inverted U-shaped relationship with albumin, with short or long sleep duration associated with significantly lower albumin levels.

**Supplementary Information:**

The online version contains supplementary material available at 10.1186/s12889-022-13524-y.

## Background

Human serum albumin is an important carrier protein and drug binding protein for various substances in plasma that possesses multiple functions, including endogenous and exogenous molecule binding and, transport, antioxidant activity, inflammatory responses, and immune modulation [1, 2]. Additionally, albumin is essential in plasma for maintaining the osmotic pressure of the blood [3]. Albumin levels are clinically useful in assessing liver function, kidney function, and nutritional status [4]. A retrospective study showed that hypoalbuminemia in patients with coronavirus disease 2019 was associated with illness progression to more severe stages and increased mortality [5]. Additionally, a recent study found that among patients with sepsis, the sleep deprivation group had significantly lower albumin than the good sleep group [6].

Sleep is critical to human health and well-being, and it is a complex and highly regulated process [7]. Serum albumin is a major antioxidant agent [8]. A study found that serum albumin levels were significantly lower in patients with sleep disorders than in those without sleep disorders [9]. The biological mechanism by which sleep duration is related to albumin levels is unclear. Among adults with objectively short sleep durations, physiological indicators of hyperarousal were increased. Increased cortisol and norepinephrine levels, result in changes in systemic metabolic rate, increased high frequency cortical dynamics, and brain glucose metabolism, and decreased gamma-aminobutyric acid levels. Regarding the effects of sleep loss on transcriptome dynamics, there is evidence that partial sleep loss during the night induces the upregulation of a gene set that includes the major circadian regulator, several immediate early genes that mark cell signaling, and multiple inflammatory response genes [10]. Albumin is the main protein synthesized in the liver and can be negatively affected by inflammation [11]. However, there is currently a limited amount of data available on the association between serum albumin levels and sleep duration. Previous studies have shown that sleep durations that are insufficient or extremely long are associated with markers of inflammation, such as C-reactive protein (CRP), CRP/ALB (CRP to albumin ratio), and the gamma gap (calculated by subtracting albumin from total protein) [12–14]. In addition, decreased albumin lipid concentrations stimulate lipoprotein production in the liver, increase blood concentrations, lead to increased peripheral vascular resistance and increased cardiovascular risk, and are associated with an increase in multiple chronic diseases, which may affect sleep.

The National Sleep Foundation (NSF) recommended 7–9 h of sleep for young adults and 7–8 h for older adults [15]. However, only 45% of individuals in the US had sleep durations that mapped onto the National Sleep Foundation recommendations on weekdays [16]. Regarding the different patterns of sleep on weekdays and weekends, a study on NHANES provided a series of benchmark estimates of sleep duration and sleep timing for the US population [17]. Shorter or longer sleep duration was linked to an increased risk of hypertension, diabetes, obesity, renal dysfunction, cancer, and mortality [18–20]. However, few epidemiological studies have assessed the relationship between sleep duration and albumin in the average adult.

Thus, we conducted this cross-sectional study to investigate the association between sleep duration and albumin levels in American adults according to NHANES.

## Methods

### Study design

NHANES is a United States national stratified multistage probability sample surveyed by the NCHS (National Center for Health Statistics) to assess nutritional status and its relationship to health promotion and disease prevention. The survey is characterized by a combination of interviews and physical examinations. Highly trained medical personnel administer the examinations and laboratory tests. Detailed descriptions of NHANES’ participant recruitment, survey design, and data collection procedures are available online at http://www.cdc.gov/nchs/nhanes/aboutnhanes.htm.

**Fig. 1:**
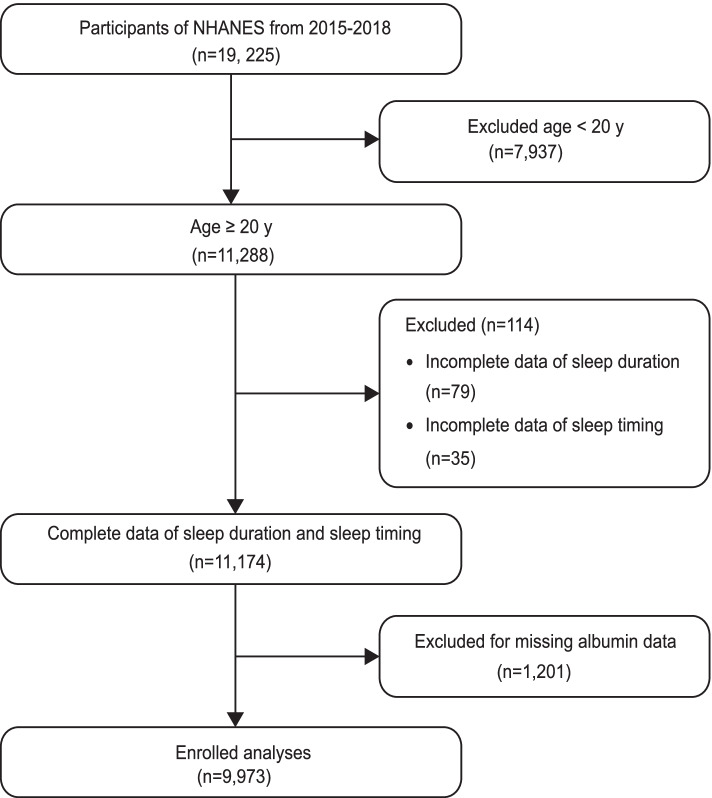
Flowchart of the participants included

This study, which used data from NHANES 2015–2018, was approved by the NCHS Research Ethics Review Board (ERB) and obtained written consent from the Centers for Disease Control and Prevention (CDC) for all surveyed individuals. Through the rigorous screening, of 19,225 participants, a total of 9,973 participants aged $\ge$ 20 years with a complete set of sleep-related data and albumin data were included in this study. The detailed screening process of participants is shown in Fig. 1.

### Measurements

#### Sleep duration and sleep timing

The definition was based on the “sleep disorders” data in the “NHANES Questionnaire”. Question SLQ012 asked “Number of hours usually sleep on weekdays or workdays.” The participants answered with their sleep duration, and hours were rounded to the nearest half-hour. Sleep duration was analysed as both a continuous and categorical variable. The sleep duration was classified and presented in 6 groups: sleep duration $\le$ 5 h, 5 h $<$ sleep duration $\le$ 6 h, 6 h $<$ sleep duration $\le$ 7 h, 7 h $<$ sleep duration $\le$ 8 h, 8 h $<$ sleep duration $\le$ 9 h, sleep duration $>$ 9 h. They are described as follows: $\le$ 5 h, 5–6 h, 6–7 h, 7–8 h, 8-9 h, $>$ 9 h [21]. (“short” sleep duration: $\le$ 5 h; “long” sleep duration: $>$ 9 h) [22, 23]. Question SLQ300 asked “ What time {do you/does SP} usually fall asleep on weekdays or workdays?” The participants answered sleep timing.

Sleep timing was recorded in 24-h notation, where HH (from 00 to 23) was the number of full hours elapsed since midnight and MM (from 00 to 59) was the number of full minutes elapsed since the last full hour. Sleep timing was divided into 4 groups: 18:00–21:59, 22:00–22:59, 23:00–23:59, and later than 00:00 [24, 25].

#### Albumin

Albumin is abbreviated as ALB. The method for measuring albumin concentration used the dye bromocresol purple. The NHANES Quality Control and Quality Assurance Protocol (QA/QC) complies with the requirements of the Clinical Laboratory Improvement Act of 1988.

#### Covariates

The covariates in this study included: demographic characteristics (age, sex, race, marital status), chronic disease (hypertension, high cholesterol, cancer or malignancy), lifestyle behavior (moderate work activity), examination data (BMI), and laboratory data (TP, ALT, AST, Cr, HS-CRP, GLU, UACR) [26].

The categorical variables among the demographic characteristics included: sex (male, female); race (Mexican American, non-Hispanic white, non-Hispanic black, other race); and marital status (married or living with a partner, living alone [never married, separated, divorced, or widowed]). Chronic diseases were assessed by self-reported medical history, and the participants were asked if they were told by a doctor or other health professional to have a specific health condition, including hypertension (yes, no, unknown), high cholesterol (yes, no, unknown), cancer or malignancy (yes, no, unknown). Moderate work activity (yes or no) was obtained from the adult section of the Physical Activity Questionnaire and categorized by answering the question, does your “work involve moderate-intensity activity that causes small increases in breathing or heart rate?” The body measurement data were collected, in the Mobile Examination Center, by trained health technicians, and BMI was calculated as weight in kilograms divided by height in meters squared. Biochemical profiles included: TP, ALT, AST, Cr, HS-CRP, GLU, and UACR. Furthermore, the estimated glomerular filtration rate (eGFR) was determined using the MDRD formula [27]: eGFR (ml/min/1.73m^2^) $=$ 175 $\times$ SCr (mg/dl)^−1.154^ $\times$ Age^−0.203^ $\times$ 0.742 (if female).

### Statistical analysis

Weighted data were calculated according to analytical guidelines (NHANES:2015–2016 and 2017–2018). Various sample weights, such as interview weight (wtint2yr), MEC (Mobile Examination Center) exam weight (wtmec2yr), and several subsample weights, are available in the NHANES data release file. The selection of the correct sample weights for the analyses depends on the variables used. All the interview and MEC exam weights covered in this study are available in the demographic files. Since the MEC examined sample persons are a subset of those interviewed in the survey, we used the combined MEC exam weight for analysis. NHANES2015-2018 involved a combination of two survey cycles (four years), and the data were weighted according to the information that the NCHS provided analysts on how to combine multiple cycles and construct appropriate weights. MEC4YR = 1/2 $\times$ WTMEC2YR [28]. Linear regression models were used to describe the relationship between sleep duration and albumin levels, and three models were constructed: Model 1, was not adjusted for any variables; Model 2, was adjusted for sex, age, race, marital status, and moderate work activity; Model 3, was adjusted for all the covariates (sex, age, race, marital status, moderate work activity, TP, ALT, AST, Cr, UACR, HS-CRP, GLU, BMI, hypertension, high cholesterol, cancer or malignancy). Smooth curve fitting was used to resolve the relationship between sleep duration and albumin. The log-likelihood ratio test was used to assess whether a threshold existed, and the inflection point was determined using a two step recursive method. We performed univariate and stratified analyses to identify independent effect between sleep duration and albumin levels. In addition, an interaction test of these factors was also performed. Continuous variables are represented by means $\pm$ SEs (standard errors), while percentages are used to represent classified variables. The effect value was expressed as β and corresponding 95% CIs (confidence intervals). *P* values $<$ 0.05 were considered statistically significant. All data in the study were analysed using the statistical package R (http://www.r-project.org; the Version: 3.4.3, 2018–02-18) and Empower Stats (http://www.empowerstats.com; X&Y Solutions Inc).

## Results

Table 1 shows the description of the characteristics of the participants grouped by sleep duration. Among the 9973 participants, 2926 slept 7–8 h, accounting for 29.3% of the total, 694 slept $\le$ 5 h, 6.96% of the total, and 1164 slept $>$ 9 h, accounting for 11.67% of the total. The total average sleep duration was 7.65 $\pm$ 1.578 h. The average ALB level was the highest in the 7–8 h group, and its highest value was 42.50 $\pm$ 3.54 g/L. Based on the sleep timing distribution, 72.83% of the participants with a sleep duration $\le$ 5 h fell asleep after 0:00.

**Table 1:** Characteristics of participants by sleep duration groups

sleep duration (h)							
	$\le$ **5**	**5–6**	**6–7**	**7–8**	**8–9**	$>$ **9**	***P* value**
N	694	1051	2248	2926	1890	1164	
sleep duration (ungrouped, h)	4.46 ± 0.67	5.89 ± 0.21	6.85 ± 0.23	7.80 ± 0.24	8.78 ± 0.25	10.28 ± 0.86	< 0.0001
sleep timing (%)							< 0.0001
18:00–21:59	2.35	7.14	11.15	17.54	28.37	38.11	
22:00–22:59	9.58	16.60	29.03	36.15	35.51	27.66	
23:00–23:59	15.25	33.20	32.32	28.50	19.45	17.22	
later than 00:00	72.83	43.06	27.50	17.82	16.67	17.01	
age (years)	49.01 ± 16.10	47.33 ± 15.13	47.36 ± 15.73	47.93 ± 16.65	49.10 ± 18.54	49.82 ± 20.32	0.0002
sex (%)							< 0.0001
male	54.43	55.38	55.74	46.80	38.49	39.59	
female	45.57	44.62	44.26	53.20	61.51	60.41	
race							< 0.0001
Mexican American	17.56	18.54	16.56	13.58	14.84	17.12	
non-Hispanic white	48.41	56.31	62.47	68.50	67.58	59.90	
non-Hispanic black	23.87	15.92	10.56	7.89	8.20	13.83	
other race	10.16	9.23	10.41	10.03	9.38	9.15	
marital status (%)							< 0.0001
married or living with partner	52.72	63.89	67.28	67.04	63.62	52.60	
living alone	47.28	36.11	32.72	32.96	36.38	47.40	
moderate work activity (%)							< 0.0001
yes	51.69	54.68	47.91	47.82	45.29	37.73	
no	48.31	45.32	52.09	52.18	54.71	62.27	
TP (g/L)	71.68 ± 4.57	70.94 ± 4.39	71.25 ± 4.29	70.97 ± 4.19	70.75 ± 4.46	71.12 ± 4.61	< 0.0001
ALT (IU/L)	26.43 ± 21.66	25.58 ± 16.57	24.92 ± 16.58	24.01 ± 16.41	23.43 ± 16.29	22.40 ± 15.94	< 0.0001
AST (IU/L)	25.63 ± 18.35	24.23 ± 12.30	24.06 ± 13.01	23.62 ± 10.20	23.68 ± 11.96	23.57 ± 13.98	0.0135
Crlog_2_ (μmol/L)	6.25 ± 0.40	6.23 ± 0.38	6.23 ± 0.36	6.20 ± 0.37	6.18 ± 0.38	6.23 ± 0.46	< 0.0001
UACRlog_2_ (mg/g)	3.26 ± 1.52	3.07 ± 1.45	3.07 ± 1.56	3.08 ± 1.47	3.14 ± 1.41	3.51 ± 1.78	< 0.0001
HS-CRP (mg/L)	3.65 ± 4.55	4.38 ± 8.20	3.51 ± 5.98	3.86 ± 7.47	3.81 ± 6.31	4.84 ± 10.44	< 0.0001
GLU (mmol/L)	5.71 ± 2.09	5.63 ± 1.87	5.55 ± 1.75	5.52 ± 1.90	5.52 ± 1.71	5.73 ± 2.02	0.0079
BMI (kg/m^2^)	30.42 ± 7.62	30.52 ± 7.52	29.84 ± 7.14	29.27 ± 6.67	29.58 ± 7.20	29.22 ± 7.02	< 0.0001
hypertension							< 0.0001
yes	40.07	34.95	30.46	30.79	31.12	38.41	
no	59.78	65.05	69.45	69.13	68.73	61.43	
unknown	0.15		0.09	0.08	0.15	0.16	
high cholesterol (%)							< 0.0001
yes	34.32	33.75	31.49	33.10	34.84	36.24	
no	64.71	65.69	68.20	66.80	64.78	62.62	
unknown	0.97	0.56	0.31	0.11	0.38	1.14	
cancer or malignancy (%)							< 0.0001
yes	9.26	8.44	8.85	10.38	13.88	13.19	
no	90.74	91.28	91.13	89.62	85.96	86.81	
unknown		0.27	0.03		0.16		
ALB (g/L)	41.41 ± 3.48	41.81 ± 3.44	42.49 ± 3.68	42.50 ± 3.54	42.04 ± 3.67	41.66 ± 3.98	< 0.0001

N referred to sample size

Mean ± SEs for continuous variables, *P* values were calculated by weighted linear regression model

Percentage (%) for categorical variables, *P* values were calculated by weighted chi-square test

*Abbreviations:* *ALB* Albumin, *TP* Total Protein, *ALT* Alanine aminotransferase, *AST* Aspartate aminotransferase, *Cr* Creatinine, *HS-CRP* High Sensitivity C-reactive Protein, *GLU* Glucose, *BMI* Body Mass Index, *UACR* Urinary Albumin-creatinine Ratio

UACR and Cr were estimated after logarithmic transformation

**Fig. 2:**
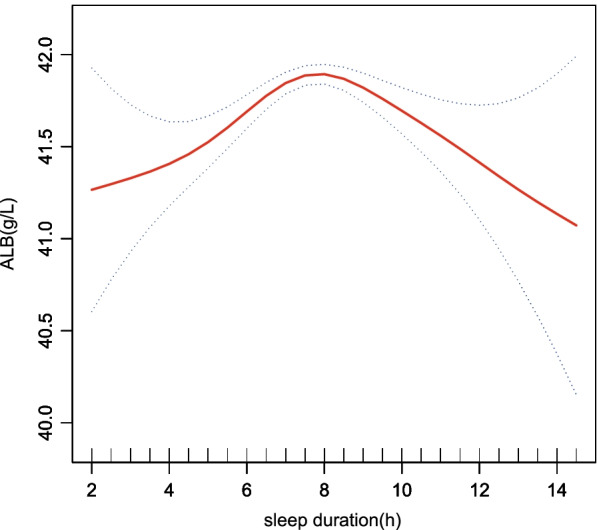
A threshold, nonlinear association between sleep duration and albumin levels was found in a generalized additive model (GAM). The solid red line represents the smooth curve fit between variables. Blue bands represent the 95% confidence interval around the fit. The model was adjusted for sex, age, race, marital status, moderate work activity, TP, ALT, AST, Cr, UACR, HS-CRP, GLU, BMI, hypertension, high cholesterol, cancer or malignancy

Sleep duration and albumin (ALB) data were grouped by sleep duration, and the relationship between covariates and ALB was analysed through univariate analyses, as shown in Supplemental Table S1. Table 2 shows that in multivariable regression models, ALB levels in those with short-sleep or long-sleep durations was lower than that in those with sleep durations of 7–8 h. In Model 3, which was adjusted for all covariates, when the sleep duration of the participants was $\le$ 5 h, it can be concluded that the effect size was β = -1.00, the 95% confidence interval was (-1.26, -0.74), and the *p* value was less than 0.0001. Similarly, when the sleep duration of participants was $>$ 9 h, conclusions can be drawn that the effect size was β $=$-0.48, the 95% confidence interval was (-0.68, -0.27), and the *p* value was less than 0.0001. Table 2 also shows that compared with the ALB levels of the participants who fell asleep between 22:00 and 22:59, ALB levels showed a more significant decrease after 00:00. In Model 3, compared with the ALB levels of the participants who fell asleep between 22:00–22:59, the ALB levels in the participants sleeping after 00:00 decreased (β, -0.43; 95% CI, -0.59, -0.28; *P* < 0.0001). An inverted U-shaped association between sleep duration and albumin was found in the GAM (generalized additive model), as presented in Fig. 2. where the solid line represents a smooth curve fit between the variables, and the blue bars represent the 95% confidence interval for the fit. We adjusted for all covariates (sex, age, race, marital status, moderate work activity, TP, ALT, AST, Cr, UACR, HS-CRP, GLU, BMI, hypertension, high cholesterol, and cancer or malignancy). Furthermore, we performed a log-likelihood ratio test and then compared the one-line linear regression model with the two-segment regression model. The inflection point K $=$ 7.5 h was obtained through a two-step recursive method. The results showed that ALB levels were highest when the sleep duration was 7.5 h. The results are shown in Table 3.

**Table 2:** Relationship between sleep duration/ sleep onset timing and albumin in different models

	**Model 1**		**Model 2**		**Model 3**	
	**β(95%CI)**	***P* value**	**β(95%CI)**	***P* value**	**β(95%CI)**	***P* value**
**sleep duration (h)**						
**7–8**	Ref		Ref		Ref	
** ≤ 5**	-1.09 (-1.43, -0.76)	< 0.0001	-0.93 (-1.24, -0.61)	< 0.0001	-1.00 (-1.26, -0.74)	< 0.0001
** 5–6**	-0.70 (-0.96, -0.44)	< 0.0001	-0.72 (-0.96, -0.47)	< 0.0001	-0.45 (-0.65, -0.25)	< 0.0001
** 6–7**	-0.02 (-0.21, 0.18)	0.8581	-0.14 (-0.32, 0.05)	0.1420	-0.13 (-0.28, 0.02)	0.0919
** 8–9**	-0.47 (-0.67, -0.26)	< 0.0001	-0.26 (-0.45, -0.07)	0.0079	-0.22 (-0.38, -0.06)	0.0063
** > 9**	-0.84 (-1.11, -0.58)	< 0.0001	-0.54 (-0.79, -0.30)	< 0.0001	-0.48 (-0.68, -0.27)	< 0.0001
**sleep timing (HH:MM)**						
** 22:00–22:59**	Ref		Ref		Ref	
** 18:00–21:59**	-0.35 (-0.56, -0.13)	0.0014	-0.27 (-0.46, -0.07)	0.0091	-0.16 (-0.33, 0.01)	0.0580
** 23:00–23:59**	-0.16 (-0.36, 0.03)	0.0901	-0.23 (-0.41, -0.05)	0.0128	-0.18 (-0.33, -0.03)	0.0166
** later than 00:00**	-0.35 (-0.55, -0.16)	0.0003	-0.61 (-0.80, -0.43)	< 0.0001	-0.43 (-0.59, -0.28)	< 0.0001

β was the effect size (g/L) of the change in albumin, and the 95% CI indicated the confidence interval

Model 1 adjust for: none

Model 2 adjust for: sex, age, race, marital status, moderate work activity

Model 3 adjust for: sex, age, race, marital status, moderate work activity, TP, ALT, AST, Cr, UACR, HS-CRP, GLU, BMI, hypertension, high cholesterol, cancer or malignancy

**Table 3:** Threshold effect analysis of sleep duration and albumin using piece-wise linear regression

Models	β (95%CI)	*P* value
Model I		
One line effect	0.04 (0.00, 0.08)	0.0366
Model II		
Inflection point (K)	7.5	
sleep duration $<$ 7.5	0.30 (0.23, 0.38)	$<$ 0.0001
sleep duration $\ge$ 7.5	-0.18 (-0.25, -0.12)	$<$ 0.0001
P for log-likelihood ratio test		$<$ 0.001

Model I, one-line linear regression; Model II, two-segment regression

β was the effect size and the 95% CI indicated the confidence interval

adjust for: sex, age, race, marital status, moderate work activity, TP, ALT, AST, Cr, UACR, HS-CRP, GLU, BMI, hypertension, high cholesterol, cancer or malignancy

**Fig. 3:**
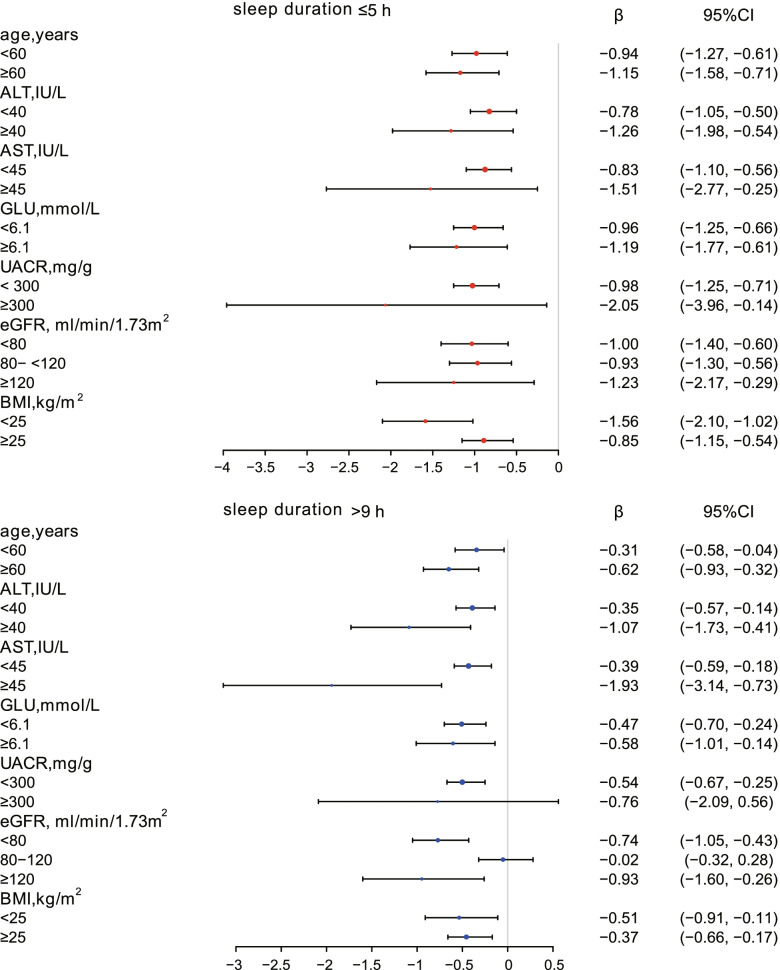
In subgroups analysed by age, ALT, AST, eGFR, UACR, GLU, and BMI, the effect size (β) and 95% confidence interval (95% CI) of changes in ALB when sleep duration ≤ 5 h and sleep duration $>$ 9 h are shown in forest plots

We evaluated the effect size of sleep duration on albumin in prespecified and exploratory subgroups. Stratified analyses and interaction tests were performed, as shown in Supplemental Table S2. Sex, race, marital status, and high cholesterol categorical variables as well as AST, and eGFR (after classification by clinical significance) had significant interactions. (P for interaction $<$ 0.05), as shown in Table 4. In terms of sex, the decreases in albumin levels were more pronounced in females than in males when sleep duration was short or long. When stratified by race, decreases in albumin levels were most marked in non-Hispanic whites with short or long sleep duration compared to albumin levels in those with 7–8 h sleep duration. When the participants slept less than 5 h, ALB decreased by 1. 67 g/L, 95% CI (-2.16, -1.17); when the participants slept more than 9 h, ALB decreased by 0.65 g/L, 95% CI (-1.00, -0.30). When stratified by marital status, the albumin of married individuals or those living with a partner fell appreciably when sleep duration was $\le$ 5 h (β, -1.67; 95% CI, -2.16, -1.17) or $>$ 9 h (β, -0.65; 95% CI, -1.00, -0.30). The participants with high cholesterol and short or long sleep durations had lower levels of ALB than those without hypercholesterolaemia. When sleep duration was $\le$ 5 h, ALB levels of hypercholesterolaemic participants decreased by 1. 23 g/L, 95% CI (-1.65, -0.81); when sleep duration was $>$ 9 h, their albumin levels decreased by 0. 65 g/L, 95% CI (-1.00, -0.30). The data for continuous variables (age, ALT, AST, eGFR, UACR, GLU, BMI) were stratified and analysed according to their clinical significance. The effect size (β) and 95% confidence interval (95% CI) of changes in ALB when sleep duration ≤ 5 h or > 9 h are shown in forest plots (Fig. 3).

**Table 4:** Effect size of sleep duration on albumin in prespecified and exploratory subgroups

sleep duration (h)							*P* for interaction
	**7–8**	$\le$ **5**	**5–6**	**6–7**	**8–9**	$>$ **9**	
**sex**							0.0418
male	Ref	-0.94 (-1.29, -0.60)	-0.45 (-0.71, -0.18)	0.09 (-0.12, 0.29)	-0.18 (-0.42, 0.06)	-0.28 (-0.59, 0.03)	
*P* value		< 0.0001	0.0011	0.4020	0.1493	0.0739	
female	Ref	-0.95 (-1.34, -0.56)	-0.47 (-0.77, -0.17)	-0.38 (-0.60, -0.15)	-0.26 (-0.47, -0.05)	-0.58 (-0.86, -0.31)	
*P* value		< 0.0001	0.0024	0.0009	0.0159	< 0.0001	
**race**							0.0010
Mexican American	Ref	-0.86 (-1.34, -0.39)	-0.40 (-0.76, -0.03)	-0.19 (-0.48, 0.10)	0.00 (-0.31, 0.32)	-0.05 (-0.43, 0.33)	
*P* value		0.0004	0.0324	0.2015	0.9940	0.7933	
non-Hispanic white	Ref	-1.67 (-2.16, -1.17)	-0.53 (-0.89, -0.18)	-0.15 (-0.41, 0.10)	-0.32 (-0.58, -0.05)	-0.65 (-1.00, -0.30)	
*P* value		< 0.0001	0.0033	0.2339	0.0182	0.0003	
non-Hispanic black	Ref	0.05 (-0.40, 0.50)	-0.13 (-0.55, 0.29)	0.02 (-0.35, 0.39)	-0.02 (-0.43, 0.40)	-0.06 (-0.50, 0.38)	
*P* value		0.8126	0.5405	0.9224	0.9433	0.7906	
other race	Ref	-0.02 (-0.67, 0.62)	-0.15 (-0.67, 0.37)	0.10 (-0.28, 0.47)	-0.18 (-0.59, 0.23)	-0.43 (-0.96, 0.10)	
*P* value		0.9466	0.5760	0.6143	0.3842	0.1095	
**marital status**							0.0023
married or living with partner	Ref	-1.38 (-1.75, -1.01)	-0.67 (-0.93, -0.41)	-0.23 (-0.42, -0.04)	-0.26 (-0.47, -0.06)	-0.69 (-0.97, -0.40)	
*P* value		< 0.0001	< 0.0001	0.0190	0.0116	< 0.0001	
living alone	Ref	-0.54 (-0.91, -0.17)	-0.06 (-0.38, 0.27)	0.08 (-0.17, 0.33)	-0.12 (-0.38, 0.13)	-0.17 (-0.46, 0.13)	
*P* value		0.0047	0.7374	0.5184	0.3454	0.2722	
**high cholesterol**							0.0177
yes	Ref	-1.23 (-1.65, -0.81)	-0.61 (-0.93, -0.28)	-0.51 (-0.76, -0.26)	-0.41 (-0.66, -0.15)	-0.83 (-1.15, -0.50)	
*P* value		< 0.0001	0.0002	< 0.0001	0.0018	< 0.0001	
no	Ref	-0.80 (-1.14, -0.46)	-0.40 (-0.66, -0.14)	0.05 (-0.14, 0.25)	-0.14 (-0.34, 0.07)	-0.28 (-0.55, -0.02)	
*P* value		< 0.0001	0.0023	0.5709	0.1879	0.0367	
**AST, IU/L**							0.0004
< 45	Ref	-0.83 (-1.10, -0.56)	-0.50 (-0.71, -0.30)	-0.12 (-0.27, 0.03)	-0.21 (-0.37, -0.05)	-0.39 (-0.59, -0.18)	
*P* value		< 0.0001	< 0.0001	0.1197	0.0104	0.0003	
≥ 45	Ref	-1.51 (-2.77, -0.25)	0.82 (-0.33, 1.97)	-0.18 (-1.11, 0.74)	-0.20 (-1.18, 0.79)	-1.93 (-3.14, -0.73)	
		0.0190	0.1629	0.7016	0.6945	0.0018	
**eGFR, ml/min/1.73m**^**2**^							0.0019
< 80	Ref	-1.00 (-1.40, -0.60)	-0.66 (-0.99, -0.33)	-0.31 (-0.55, -0.06)	-0.40 (-0.66, -0.15)	-0.74 (-1.05, -0.43)	
*P* value		< 0.0001	< 0.0001	0.0134	0.0018	< 0.0001	
80–120	Ref	-0.93 (-1.30, -0.56)	-0.48 (-0.75, -0.21)	-0.10 (-0.30, 0.10)	-0.10 (-0.32, 0.12)	-0.02 (-0.32, 0.28)	
*P* value		< 0.0001	0.0005	0.3282	0.3589	0.8870	
≥ 120	Ref	-1.23 (-2.17, -0.29)	0.26 (-0.44, 0.96)	0.21 (-0.33, 0.76)	0.02 (-0.55, 0.59)	-0.93 (-1.60, -0.26)	
*P* value		0.0106	0.4718	0.4431	0.9469	0.0064	

Each stratification adjusted for all factors (sex, age, race, marital status, moderate work activity, TP, ALT, AST, Cr, UACR, HS-CRP, GLU, BMI, hypertension, high cholesterol, cancer or malignancy) except the stratification factor itself

## Discussion

The main aim of this study was to explore the associations of sleep duration with ALB levels in a nationally representative sample of American adults. The results of this study showed that sleep duration and ALB levels showed an inverted U-shaped relationship. It was demonstrated that ALB levels were highest when the sleep duration was 7.5 h, which was in the 7–8 h group (7 h $<$ sleep duration $\le$ 8 h). When sleep duration was $\le$ 5 h (“short” sleep duration) or sleep duration was $>$ 9 h (“long” sleep duration), the albumin levels significantly decreased, so we performed a focused comparative analysis of 3 sleep duration categories (sleep duration $\le$ 5 h, 7 h $<$ sleep duration $\le$ 8 h, sleep duration $>$ 9 h).

Short sleep duration or long sleep duration was significantly associated with lower ALB levels than 7–8 h sleep duration even after adjusting for confounding factors. An animal experimental study found that sleep deprivation resulted in significantly lower serum albumin levels [29]. Albumin is an important indicator for evaluating nutritional status, and sleep duration $<$ 6 h or $>$ 9 h was indicated to increase the malnutrition risk of older individuals [21].

We evaluated the effect size of different sleep duration groups on albumin levels in prespecified and exploratory subgroups and the results showed differences. There was a significant correlation between sleep duration and ALB levels in patients with renal insufficiency, and the reason was considered that urinary albumin causes albumin levels to decrease. A cross-sectional study from the KNHANES confirmed the U-shaped relationship between sleep duration and urinary albumin in Korean adults [30]. Stratified and interaction analyses showed that sleep duration had a greater impact on albumin levels in patients with abnormal liver function. Albumin is synthesized by the liver, and impairments in liver function can lead to the blockage of albumin synthesis. ALB plays a crucial role in immunomodulation during chronic liver disease processes, and albumin is negatively correlated with infection in patients with chronic liver diseases [31]. The participants with high cholesterol or high blood sugar had more significant changes in their ALB levels with short or long sleep duration. A NHANES study showed a significant U-shaped relationship between sleep duration and metabolic syndrome severity score [32]. Circadian misalignment leads to an increased risk of metabolic disease in individuals with late sleep time [33]. Sleeping late can disrupt the rhythm of the body’s biological clock. We also found that ALB levels in those with late bedtimes were lower than in those falling asleep at 22:00–22:59, especially when the sleep timing was later than 0:00. A recent study showed that delayed sleep is the cause of short sleep duration [17]. Additionally, a study in the American population found that sleep duration had a U-shaped association with leading chronic diseases [34]. Chronic diseases often lead to a decline in the body’s immunity and nutritional status, which in turn results in a decline in albumin levels.

This study has some significance. First, our findings can provide supportive evidence to clinical work. The results of this study are consistent with NSF recommendations regarding healthy sleep durations. For adults with low albumin levels or hypoalbuminemia, especially those with chronic diseases, the sleep timing should be adjusted to ensure the optimal sleep duration. Second, to our knowledge, this is the first study to study the association between sleep duration and album levels in US adults. In addition, our findings revealed potential public health concern. We found racial differences in sleep duration, racial/ethnic minorities, particularly non-Hispanic blacks, reported shorter sleep durations than non-Hispanic whites. This is consistent with the findings of a recent study showing significant differences in sleep duration by race and ethnicity, with the incidence of unrecommended sleep durations consistently higher in black individuals [35]. These persistent disparities may lead to other persistent racial and ethnic disparities in health.

The study had certain limitations. First, due to the cross-sectional study design, causal associations cannot be established between sleep information and ALB levels. Future longitudinal studies could improve the reliability of the findings. Second, sleep duration and sleep timing were obtained from self-reported information. Studies have shown that self-reported or perceived sleep time is different from objective assessments of sleep duration accomplished by actigraphy [36, 37]. In addition, recall bias and seasonal variations may lead to information bias in the reporting of sleep duration [38]. However, in large population-based studies, self-reported data allow us to obtain more representative population estimates. Although validated sleep instruments are considered to provide objective indicators for the measurement of sleep architecture, self-reported inventories are often used to measure sleeping health owing to the advantages of being affordable, quick, and feasible to administer in large samples [39]. Finally, there might be other mixed factors that were not adequately taken into account. Despite these limitations, this is a population-based study looking at the relationship between sleep duration and ALB levels in US adults.

## Conclusions

This study shows that sleep duration has an inverted U-shaped relationship with albumin levels, in a nationally representative sample of American adults. ALB levels were highest when the sleep duration was 7.5 h. Short or long sleep durations were significantly associated with lower ALB levels compared with sleep durations of 7–8 h.

## Supplementary Information

**Additional file 1:** **Table S1.** Univariate analysis for albumin(g/L).

**Additional file 2:** **Table S2.** Effect size of sleep duration on albumin in prespecified and exploratory subgroups.

## Data Availability

The datasets generated and analysed for the current study are available in the NHANES repository. These data can be accessed using the following link: https://wwwn.cdc.gov/nchs/nhanes/Default.aspx.

## Abbreviations

NHANES: National Health and Nutrition Examination Survey
NSF: National Sleep Foundation
NCHS: National Center for Health Statistics
ERB: Ethics Review Board
CDC: Centers for Disease Control and Prevention
QA/QC: Quality Control and Quality Assurance Protocol
MEC: Mobile Examination Center
95% CI: 95% Confidence interval
ALB: Albumin
TP: Total protein
ALT: Alanine aminotransferase
AST: Aspartate aminotransferase
Cr: Creatinine
HS-CRP: High sensitivity C-reactive protein
GLU: Glucose
BMI: Body mass index
UACR: Urinary albumin-creatinine ratio
eGFR: Estimated glomerular filtration rate
