# Supplementary material for: Association between sleep duration and albumin in US adults: a cross-sectional study of NHANES 2015–2018
Source: BMC Public Health. 2022 Jun 2;22:1102. doi: 10.1186/s12889-022-13524-y (PMC9161202; doi:10.1186/s12889-022-13524-y)
Supplement: Supplementary file 1 — Additional file 1: Table S1. Univariate analysis for albumin(g/L). [file 12889_2022_13524_MOESM1_ESM.docx]

**Table S1 Univariate analysis for albumin(g/L)**

| **sleep duration (h)** | | | | | | |
| --- | --- | --- | --- | --- | --- | --- |
|  | $\boldsymbol{\leq}$**5** | **5-6** | **6-7** | **7-8** | **8-9** | $\boldsymbol{>}$**9** |
| **sex** | | | | | | |
| male | Ref | | | | | |
| female | -2.02 (-2.52, -1.52) | -2.09 (-2.49, -1.69) | -2.22 (-2.52, -1.93) | -1.72 (-1.97, -1.47) | -1.65 (-1.98, -1.31) | -1.95 (-2.40, -1.49) |
| P value | <0.0001 | <0.0001 | <0.0001 | <0.0001 | <0.0001 | <0.0001 |
| **age** | -0.03 (-0.05, -0.02) | -0.03 (-0.04, -0.01) | -0.06 (-0.06, -0.05) | -0.03 (-0.04, -0.02) | -0.02 (-0.03, -0.02) | -0.05 (-0.06, -0.03) |
| P value | <0.0001 | <0.0001 | <0.0001 | <0.0001 | <0.0001 | <0.0001 |
| **race** | | | | | | |
| non-Hispanic white | Ref | | | | | |
| non-Hispanic black | -0.08 (-0.72, 0.57) | -1.19 (-1.77, -0.60) | -1.76 (-2.26, -1.26) | -1.78 (-2.25, -1.30) | -1.35 (-1.96, -0.74) | -1.09 (-1.77, -0.41) |
| P value | 0.8097 | <0.0001 | <0.0001 | <0.0001 | <0.0001 | 0.0017 |
| Mexican American | 0.02 (-0.69, 0.74) | -0.02 (-0.58, 0.53) | -0.92 (-1.34, -0.51) | -0.62 (-1.00, -0.24) | -0.49 (-0.96, -0.01) | -0.29 (-0.92, 0.33) |
| P value | 0.9467 | 0.9334 | <0.0001 | 0.0013 | 0.0437 | 0.3588 |
| other race | 1.27 (0.38, 2.16) | 0.11 (-0.62, 0.84) | -0.41 (-0.91, 0.10) | -0.49 (-0.92, -0.06) | -0.19 (-0.76, 0.39) | 0.06 (-0.75, 0.87) |
| P value | 0.0054 | 0.7646 | 0.1126 | 0.0247 | 0.5209 | 0.8847 |
| **marital status** | | | | | | |
| living alone | Ref | | | | | |
| married or living with partner | -0.21 (-0.73, 0.31) | -0.10 (-0.53, 0.33) | -0.16 (-0.48, 0.17) | 0.31 (0.04, 0.58) | 0.05 (-0.29, 0.39) | -0.51 (-0.96, -0.05) |
| P value | 0.4244 | 0.6562 | 0.3434 | 0.0257 | 0.7777 | 0.0307 |
| **moderate work activity** | | | | | | |
| no | Ref | | | | | |
| yes | 0.11 (-0.41, 0.63) | 0.40 (-0.01, 0.82) | 0.26 (-0.05, 0.56) | 0.26 (0.01, 0.52) | 0.38 (0.05, 0.71) | 0.51 (0.04, 0.98) |
| P value | 0.6827 | 0.0582 | 0.0960 | 0.0449 | 0.0250 | 0.0331 |
| **TP (g/L)** | 0.32 (0.27, 0.37) | 0.35 (0.30, 0.39) | 0.33 (0.30, 0.36) | 0.36 (0.34, 0.39) | 0.39 (0.35, 0.42) | 0.42 (0.37, 0.46) |
| P value | <0.0001 | <0.0001 | <0.0001 | <0.0001 | <0.0001 | <0.0001 |
| **ALT (IU/L)** | 0.04 (0.03, 0.05) | 0.02 (0.01, 0.04) | 0.03 (0.02, 0.04) | 0.03 (0.02, 0.04) | 0.03 (0.02, 0.04) | 0.03 (0.01, 0.04) |
| P value | <0.0001 | <0.0001 | <0.0001 | <0.0001 | <0.0001 | 0.0002 |
| **AST (IU/L)** | 0.04 (0.03, 0.05) | 0.04 (0.02, 0.06) | 0.04 (0.03, 0.05) | 0.06 (0.05, 0.07) | 0.05 (0.03, 0.06) | 0.00 (-0.01, 0.02) |
| P value | <0.0001 | <0.0001 | <0.0001 | <0.0001 | <0.0001 | 0.7393 |
| **Crlog_2_ (μmol/L)** | 0.18 (-0.47, 0.84) | 0.82 (0.27, 1.37) | 1.30 (0.88, 1.71) | 1.18 (0.83, 1.52) | 0.71 (0.28, 1.15) | 0.50 (0.01, 1.00) |
| P value | 0.5852 | 0.0034 | <0.0001 | <0.0001 | 0.0013 | 0.0460 |
| **UACRlog_2_ (mg/g)** | -0.30 (-0.47, -0.14) | -0.37 (-0.51, -0.23) | -0.25 (-0.35, -0.16) | -0.32 (-0.40, -0.23) | -0.23 (-0.35, -0.11) | -0.31 (-0.43, -0.18) |
| P value | 0.0004 | <0.0001 | <0.0001 | <0.0001 | 0.0001 | <0.0001 |
| **HS-CRP (mg/L)** | -0.20 (-0.26, -0.15) | -0.10 (-0.13, -0.08) | -0.20 (-0.22, -0.17) | -0.13 (-0.15, -0.12) | -0.15 (-0.18, -0.13) | -0.11 (-0.13, -0.09) |
| P value | <0.0001 | <0.0001 | <0.0001 | <0.0001 | <0.0001 | <0.0001 |
| **GLU (mmol/L)** | -0.13 (-0.25, -0.01) | -0.17 (-0.28, -0.06) | -0.31 (-0.40, -0.23) | -0.27 (-0.34, -0.20) | -0.15 (-0.25, -0.06) | -0.24 (-0.36, -0.13) |
| P value | 0.0386 | 0.0028 | <0.0001 | <0.0001 | 0.0019 | <0.0001 |
| **BMI (kg/m^2^)** | -0.10 (-0.13, -0.07) | -0.15 (-0.17, -0.12) | -0.15 (-0.17, -0.13) | -0.14 (-0.16, -0.12) | -0.12 (-0.14, -0.09) | -0.15 (-0.18, -0.12) |
| P value | <0.0001 | <0.0001 | <0.0001 | <0.0001 | <0.0001 | <0.0001 |
| **hypertension** | | | | | | |
| no | Ref | | | | | |
| yes | -0.08 (-0.61, 0.45) | -0.65 (-1.09, -0.22) | -0.71 (-1.04, -0.38) | -0.83 (-1.11, -0.55) | -0.34 (-0.69, 0.02) | -1.19 (-1.65, -0.72) |
| P value | 0.7618 | 0.0034 | <0.0001 | <0.0001 | 0.0656 | <0.0001 |
| unknown | 2.56 (-4.05, 9.17) |  | -7.71(-12.73, -2.69) | 0.54 (-3.85, 4.92) | 0.45 (-3.87, 4.77) | -1.73 (-7.33, 3.88) |
| P value | 0.4480 |  | 0.0027 | 0.8110 | 0.8380 | 0.5454 |
| **high cholesterol** | | | | | | |
| no | Ref | | | | | |
| yes | -0.32 (-0.87, 0.23) | 0.08 (-0.36, 0.52) | -0.61 (-0.94, -0.28) | 0.05 (-0.22, 0.32) | -0.21 (-0.55, 0.14) | -0.74 (-1.22, -0.27) |
| P value | 0.2499 | 0.7160 | 0.0003 | 0.7144 | 0.2428 | 0.0022 |
| unknown | -1.26 (-3.92, 1.39) | 0.99 (-1.79, 3.77) | -1.87 (-4.62, 0.88) | -1.86 (-5.78, 2.07) | 0.74 (-1.94, 3.42) | -0.23 (-2.38, 1.93) |
| P value | 0.3514 | 0.4861 | 0.1836 | 0.3535 | 0.5885 | 0.8364 |
| **Cancer or malignancy** | | | | | | |
| no | Ref | | | | | |
| yes | -0.64 (-1.53, 0.25) | 0.41 (-0.33, 1.15) | -0.88 (-1.42, -0.35) | -0.62 (-1.04, -0.20) | -0.67 (-1.14, -0.19) | -1.11 (-1.78, -0.43) |
| P value | 0.1610 | 0.2794 | 0.0013 | 0.0038 | 0.0063 | 0.0013 |
| unknown |  | -7.30(-11.27, -3.33) | -5.76(-14.97, 3.44) |  | -2.53 (-6.61, 1.55) |  |
| P value |  | 0.0003 | 0.2197 |  | 0.2247 |  |

The values in the table were expressed as β (95% CI), where β was the effect size (g/L) of the change in albumin, and the 95%CI indicated the confidence interval.

Abbreviations: TP, total protein; ALT, alanine aminotransferase; AST, aspartate aminotransferase; Cr, creatinine; UACR, urinary albumin-creatinine ratio; HS-CRP, high sensitivity C-reactive Protein; GLU, glucose; BMI, body mass index.
