# Supplementary material for: Association between sleep duration and albumin in US adults: a cross-sectional study of NHANES 2015–2018
Source: BMC Public Health. 2022 Jun 2;22:1102. doi: 10.1186/s12889-022-13524-y (PMC9161202; doi:10.1186/s12889-022-13524-y)
Supplement: Supplementary file 2 — Additional file 2: Table S2. Effect size of sleep duration on albumin in prespecified and exploratory subgroups. [file 12889_2022_13524_MOESM2_ESM.docx]

**Table S2 Effect size of sleep duration on albumin in prespecified and exploratory subgroups**

| **sleep duration (h)** | | | | | | | **P for interaction** |
| --- | --- | --- | --- | --- | --- | --- | --- |
|  | **7-8** | $\boldsymbol{\leq}$**5** | **5-6** | **6-7** | **8-9** | $\boldsymbol{>}$**9** |  |
| **sex** | | | | | | | 0.0418 |
| male | Ref | -0.94 (-1.29, -0.60) | -0.45 (-0.71, -0.18) | 0.09 (-0.12, 0.29) | -0.18 (-0.42, 0.06) | -0.28 (-0.59, 0.03) |  |
| P value |  | <0.0001 | 0.0011 | 0.4020 | 0.1493 | 0.0739 |  |
| female | Ref | -0.95 (-1.34, -0.56) | -0.47 (-0.77, -0.17) | -0.38 (-0.60, -0.15) | -0.26 (-0.47, -0.05) | -0.58 (-0.86, -0.31) |  |
| P value |  | <0.0001 | 0.0024 | 0.0009 | 0.0159 | <0.0001 |  |
| **race** | | | | | | | 0.0010 |
| Mexican American | Ref | -0.86 (-1.34, -0.39) | -0.40 (-0.76, -0.03) | -0.19 (-0.48, 0.10) | 0.00 (-0.31, 0.32) | -0.05 (-0.43, 0.33) |  |
| P value |  | 0.0004 | 0.0324 | 0.2015 | 0.9940 | 0.7933 |  |
| non-Hispanic white | Ref | -1.67 (-2.16, -1.17) | -0.53 (-0.89, -0.18) | -0.15 (-0.41, 0.10) | -0.32 (-0.58, -0.05) | -0.65 (-1.00, -0.30) |  |
| P value |  | <0.0001 | 0.0033 | 0.2339 | 0.0182 | 0.0003 |  |
| non-Hispanic black | Ref | 0.05 (-0.40, 0.50) | -0.13 (-0.55, 0.29) | 0.02 (-0.35, 0.39) | -0.02 (-0.43, 0.40) | -0.06 (-0.50, 0.38) |  |
| P value |  | 0.8126 | 0.5405 | 0.9224 | 0.9433 | 0.7906 |  |
| other race | Ref | -0.02 (-0.67, 0.62) | -0.15 (-0.67, 0.37) | 0.10 (-0.28, 0.47) | -0.18 (-0.59, 0.23) | -0.43 (-0.96, 0.10) |  |
| P value |  | 0.9466 | 0.5760 | 0.6143 | 0.3842 | 0.1095 |  |
| **marital status** | | | | | | | 0.0023 |
| married or living with partner | Ref | -1.38 (-1.75, -1.01) | -0.67 (-0.93, -0.41) | -0.23 (-0.42, -0.04) | -0.26 (-0.47, -0.06) | -0.69 (-0.97, -0.40) |  |
| P value |  | <0.0001 | <0.0001 | 0.0190 | 0.0116 | <0.0001 |  |
| living alone | Ref | -0.54 (-0.91, -0.17) | -0.06 (-0.38, 0.27) | 0.08 (-0.17, 0.33) | -0.12 (-0.38, 0.13) | -0.17 (-0.46, 0.13) |  |
| P value |  | 0.0047 | 0.7374 | 0.5184 | 0.3454 | 0.2722 |  |
| **moderate work activity** | | | | | | | 0.5889 |
| yes | Ref | -1.17 (-1.56, -0.77) | -0.55 (-0.85, -0.26) | -0.16 (-0.40, 0.07) | -0.22 (-0.47, 0.04) | -0.45 (-0.80, -0.10) |  |
| P value |  | <0.0001 | 0.0003 | 0.1766 | 0.0933 | 0.0117 |  |
| no | Ref | -0.72 (-1.07, -0.36) | -0.35 (-0.63, -0.06) | -0.11 (-0.31, 0.09) | -0.21 (-0.41, -0.00) | -0.46 (-0.71, -0.21) |  |
| P value |  | <0.0001 | 0.0159 | 0.2888 | 0.0484 | 0.0003 |  |
| **hypertension** | | | | | | | 0.1867 |
| yes | Ref | -0.74 (-1.15, -0.33) | -0.24 (-0.58, 0.09) | 0.00 (-0.27, 0.27) | 0.10 (-0.18, 0.37) | -0.40 (-0.73, -0.07) |  |
| P value |  | 0.0004 | 0.1592 | 0.9973 | 0.5044 | 0.0190 |  |
| no | Ref | -1.07 (-1.41, -0.73) | -0.54 (-0.80, -0.29) | -0.18 (-0.36, 0.01) | -0.36 (-0.56, -0.17) | -0.54 (-0.80, -0.28) |  |
| P value |  | <0.0001 | <0.0001 | 0.0616 | 0.0003 | <0.0001 |  |
| **high cholesterol** | | | | | | | 0.0177 |
| yes | Ref | -1.23 (-1.65, -0.81) | -0.61 (-0.93, -0.28) | -0.51 (-0.76, -0.26) | -0.41 (-0.66, -0.15) | -0.83 (-1.15, -0.50) |  |
| P value |  | <0.0001 | 0.0002 | <0.0001 | 0.0018 | <0.0001 |  |
| no | Ref | -0.80 (-1.14, -0.46) | -0.40 (-0.66, -0.14) | 0.05 (-0.14, 0.25) | -0.14 (-0.34, 0.07) | -0.28 (-0.55, -0.02) |  |
| P value |  | <0.0001 | 0.0023 | 0.5709 | 0.1879 | 0.0367 |  |
| **cancer or malignancy** | | | | | | | 0.1186 |
| yes | Ref | -1.15 (-2.06, -0.24) | 0.08 (-0.63, 0.79) | -0.29 (-0.81, 0.22) | -0.44 (-0.92, 0.03) | -1.04 (-1.65, -0.43) |  |
| P value |  | 0.0135 | 0.8316 | 0.2649 | 0.0691 | 0.0009 |  |
| no | Ref | -0.93 (-1.20, -0.65) | -0.48 (-0.69, -0.27) | -0.12 (-0.28, 0.04) | -0.19 (-0.36, -0.02) | -0.40 (-0.62, -0.18) |  |
| P value |  | <0.0001 | <0.0001 | 0.1470 | 0.0279 | 0.0004 |  |
| **age, years** | | | | | | | 0.1994 |
| < 60 | Ref | -0.94 (-1.27, -0.61) | -0.48 (-0.73, -0.24) | -0.13 (-0.32, 0.05) | -0.28 (-0.48, -0.08) | -0.31 (-0.58, -0.04) |  |
| P value |  | <0.0001 | 0.0001 | 0.1635 | 0.0070 | 0.0255 |  |
| ≥60 | Ref | -1.14 (-1.58, -0.71) | -0.31 (-0.68, 0.06) | -0.14 (-0.41, 0.13) | -0.05 (-0.30, 0.21) | -0.62 (-0.93, -0.32) |  |
|  |  | <0.0001 | 0.1053 | 0.2997 | 0.7088 | <0.0001 |  |
| **ALT, IU/L** | | | | | | | 0.3115 |
| < 40 | Ref | -0.78 (-1.05, -0.50) | -0.41 (-0.62, -0.19) | -0.10 (-0.26, 0.06) | -0.23 (-0.39, -0.06) | -0.35 (-0.57, -0.14) |  |
| P value |  | <0.0001 | 0.0002 | 0.2114 | 0.0071 | 0.0011 |  |
| ≥40 | Ref | -1.26 (-1.98, -0.54) | -0.57 (-1.13, -0.00) | -0.16 (-0.63, 0.30) | -0.19 (-0.73, 0.35) | -1.07 (-1.73, -0.41) |  |
| P value |  | 0.0006 | 0.0501 | 0.4871 | 0.4909 | 0.0016 |  |
| **AST, IU/L** | | | | | | | 0.0004 |
| < 45 | Ref | -0.83 (-1.10, -0.56) | -0.50 (-0.71, -0.30) | -0.12 (-0.27, 0.03) | -0.21 (-0.37, -0.05) | -0.39 (-0.59, -0.18) |  |
| P value |  | <0.0001 | <0.0001 | 0.1197 | 0.0104 | 0.0003 |  |
| ≥45 | Ref | -1.51 (-2.77, -0.25) | 0.82 (-0.33, 1.97) | -0.18 (-1.11, 0.74) | -0.20 (-1.18, 0.79) | -1.93 (-3.14, -0.73) |  |
|  |  | 0.0190 | 0.1629 | 0.7016 | 0.6945 | 0.0018 |  |
| **eGFR, ml/min/1.73m^2^** | | | | | | | 0.0019 |
| < 80 | Ref | -1.00 (-1.40, -0.60) | -0.66 (-0.99, -0.33) | -0.31 (-0.55, -0.06) | -0.40 (-0.66, -0.15) | -0.74 (-1.05, -0.43) |  |
| P value |  | <0.0001 | <0.0001 | 0.0134 | 0.0018 | <0.0001 |  |
| 80-120 | Ref | -0.93 (-1.30, -0.56) | -0.48 (-0.75, -0.21) | -0.10 (-0.30, 0.10) | -0.10 (-0.32, 0.12) | -0.02 (-0.32, 0.28) |  |
| P value |  | <0.0001 | 0.0005 | 0.3282 | 0.3589 | 0.8870 |  |
| ≥120 | Ref | -1.23 (-2.17, -0.29) | 0.26 (-0.44, 0.96) | 0.21 (-0.33, 0.76) | 0.02 (-0.55, 0.59) | -0.93 (-1.60, -0.26) |  |
| P value |  | 0.0106 | 0.4718 | 0.4431 | 0.9469 | 0.0064 |  |
| **UACR, mg/g** | | | | | | | 0.3911 |
| < 300 | Ref | -0.98 (-1.25, -0.71) | -0.45 (-0.66, -0.25) | -0.14 (-0.29, 0.01) | -0.24 (-0.40, -0.08) | -0.46 (-0.67, -0.25) |  |
| P value |  | <0.0001 | <0.0001 | 0.0713 | 0.0038 | <0.0001 |  |
| ≥300 | Ref | -2.05 (-3.96, -0.14) | -0.55 (-2.41, 1.30) | -0.22 (-1.48, 1.05) | 0.92 (-0.55, 2.38) | -0.76 (-2.09, 0.56) |  |
| P value |  | 0.0367 | 0.5612 | 0.7389 | 0.2205 | 0.2596 |  |
| **GLU, mmol/L** | | | | | | | 0.2184 |
| < 6.1 | Ref | -0.96 (-1.25, -0.66) | -0.46 (-0.68, -0.23) | -0.05 (-0.22, 0.12) | -0.16 (-0.34, 0.02) | -0.47 (-0.70, -0.24) |  |
| P value |  | <0.0001 | <0.0001 | 0.5585 | 0.0771 | <0.0001 |  |
| ≥6.1 | Ref | -1.19 (-1.77, -0.61) | -0.42 (-0.88, 0.03) | -0.54 (-0.89, -0.19) | -0.52 (-0.89, -0.15) | -0.58 (-1.01, -0.14) |  |
| P value |  | <0.0001 | 0.0679 | 0.0026 | 0.0054 | 0.0093 |  |
| **BMI, kg/m^2^** | | | | | | | 0.2036 |
| < 25 | Ref | -1.56 (-2.10, -1.02) | -0.63 (-1.05, -0.21) | -0.13 (-0.43, 0.17) | -0.22 (-0.53, 0.08) | -0.51 (-0.91, -0.11) |  |
| P value |  | <0.0001 | 0.0030 | 0.3911 | 0.1503 | 0.0132 |  |
| ≥25 | Ref | -0.85 (-1.15, -0.54) | -0.45 (-0.68, -0.21) | -0.17 (-0.35, 0.01) | -0.28 (-0.47, -0.09) | -0.42 (-0.66, -0.17) |  |
| P value |  | <0.0001 | 0.0002 | 0.0603 | 0.0045 | 0.0009 |  |

Each stratification adjusted for all factors (sex, age, race, marital status, moderate work activity, TP, ALT, AST, Cr, UACR, HS-CRP, GLU, BMI, hypertension, high cholesterol, cancer or malignancy) except the stratification factor itself.
